# Genetic and Epigenomic Modifiers of Diabetic Neuropathy

**DOI:** 10.3390/ijms22094887

**Published:** 2021-05-05

**Authors:** Milena Jankovic, Ivana Novakovic, Dejan Nikolic, Jasmina Mitrovic Maksic, Slavko Brankovic, Ivana Petronic, Dragana Cirovic, Sinisa Ducic, Mirko Grajic, Dragana Bogicevic

**Affiliations:** 1 Neurology Clinic, Clinical Center of Serbia, 11000 Belgrade, Serbia; milena.jankovic.82@gmail.com; 2 Faculty of Medicine, University of Belgrade, 11000 Belgrade, Serbia; ivana.novakovic@med.bg.ac.rs (I.N.); denikol27@gmail.com (D.N.); ivana.pm@live.com (I.P.); dragana.cirovic@udk.bg.ac.rs (D.C.); sinisa.ducic@med.bg.ac.rs (S.D.); mirko.grajic@med.bg.ac.rs (M.G.); 3 Physical Medicine and Rehabilitation Department, University Children’s Hospital, 11000 Belgrade, Serbia; 4 Faculty of Special Education and Rehabilitation, University of Belgrade, 11000 Belgrade, Serbia; dejan.nikolic@med.bg.ac.rs; 5 Department of Biology, Faculty of Sciences and Mathematics, University in Pristina-Kosovska Mitrovica, 38220 Kosovska Mitrovica, Serbia; slavko.brankovic@pr.ac.rs; 6 Pediatric Surgery Department, University Children’s Hospital, 11000 Belgrade, Serbia; 7 Physical Medicine and Rehabilitation Clinic, Clinical Center of Serbia, 11000 Belgrade, Serbia; 8 Pediatric Department, University Children’s Hospital, 11000 Belgrade, Serbia

**Keywords:** diabetic neuropathy, genetic markers, gene polymorphisms, epigenetics

## Abstract

Diabetic neuropathy (DN), the most common chronic and progressive complication of diabetes mellitus (DM), strongly affects patients’ quality of life. DN could be present as peripheral, autonomous or, clinically also relevant, uremic neuropathy. The etiopathogenesis of DN is multifactorial, and genetic components play a role both in its occurrence and clinical course. A number of gene polymorphisms in candidate genes have been assessed as susceptibility factors for DN, and most of them are linked to mechanisms such as reactive oxygen species production, neurovascular impairments and modified protein glycosylation, as well as immunomodulation and inflammation. Different epigenomic mechanisms such as DNA methylation, histone modifications and non-coding RNA action have been studied in DN, which also underline the importance of “metabolic memory” in DN appearance and progression. In this review, we summarize most of the relevant data in the field of genetics and epigenomics of DN, hoping they will become significant for diagnosis, therapy and prevention of DN.

## 1. Introduction

Diabetes mellitus (DM) presents a worldwide public health burden. It is associated with numerous complications, including diabetic neuropathy (DN), which occurs in around 60–70% of affected individuals [1]. Furthermore, it was stated that the prevalence of DN in newly diagnosed patients with DM is around 8% [2]. The DN can be presented as peripheral diabetic neuropathy (PDN) as well as diabetic autonomic neuropathy (DAN) [3]. Additionally, from the clinical perspective, DN can be presented as uremic neuropathy (UN) [1].

The etiopathogenesis of DN is multifactorial and involves environmental, external factors and internal factors [1,2,4,5]. In the meta-analysis of Wu et al., it was further stated that genetic factors might influence the course of PDN [4]. Moreover, in the study of Kallinikou et al., it was stressed that subclinical DN can be present in the pediatric population even with good metabolic control and short DM duration, which might suggest potential genetic susceptibility [3].

The role of heritability in DN has been assumed based on the results of twin studies and observed familial aggregation [1]. In contemporary studies, medical genetics main tools for assessment of genetic predisposition to multifactorial diseases are association studies. Such studies analyze variants known as DNA/gene polymorphisms with the aim to detect if a particular variant is significantly linked to disease occurrence, representing a genetic risk factor. Protective variants which are less frequent in affected persons could be detected also. Common association studies analyze variants in a small number of candidate genes, which are selected by known patho-physiological mechanisms, by results in animal models or in segregation studies. In the last two decades, Genome Wide Association Studies (GWAS) are available, which allow simultaneous analysis of thousands of (unselected) DNA variants scattered throughout the entire genome. More recently, development of Next Generation Sequencing technology enabled rapid detection of DNA/gene variants contributing to genetics of multifactorial diseases also.

In the field of genetics of DN, a number of candidate gene studies have been published so far, but often with inconsistent results and with substantial missing heritability. Several issues, well known for such type of studies in general, are different sample size and structure, population and ethnic specificity, complex interaction between genes and environment, etc. [1,2,6].

In this article, first will be presented the main patho-physiological mechanisms important in DN, and then based on that, major results of association studies for candidate genes. Finally, epigenetic concepts important in DN will be discussed.

Besides the multidimensional role of different factors that could be associated with the DN, including but not limited to: reactive oxygens species production, neurovascular impairments and modified protein glycosylation, there are not sufficient findings regarding the role of inflammatory mediators in the DN etiopathogenesis [7].

## 2. Anatomical, Histological and Clinical Considerations

Peripheral diabetic neuropathy can be described as a common late complication of diabetes and is significant contributor to the increased morbidity and mortality [8,9]. It is presented with a wide plethora of abnormalities affecting motor, sensory and autonomic nerves that can be clinically expressed as isolated or combined. 

PDN is usually painful and despite the fact that several structural and functional differences were noticed in central nervous system (CNS) between painful and painless PDN, in the study of Groener et al. [9] no specific distinguishing features in peripheral nervous system (PNS) between these two groups of PDN were not noticed. Diabetic polyneuropathy is often presented as mixed fiber polyneuropathy that involves Aβ, Aδ and C-fibers, however neuropathic pain is mostly associated with a small-fiber affection, but in the study of Galosi et al. [10], neuropathic pain was described in pure large-fiber neuropathy as well. In Table 1, characteristics of different types of neuropathies are presented [11,12,13,14,15,16,17,18,19,20]. 

The complexity of diabetic autonomic neuropathy (DAN) can be found in numerous studies [12,13,14,15,16,17,18,19,20]. It was noticed that patients with DAN have changes in CNS including neuron loss in intermediolateral nuclei of the spinal cord, presence of degeneration along with the loss of sympathetic neurons of prevertebral and paravertebral autonomic ganglia, as well as vagal nerve axons degeneration, and loss of autonomic axons in somatic nerves [21].

In DN, reduction of unmyelinated and small myelinated axons was shown to appear earlier than decrease in large myelinated fibers, with assumption that changes in Schwann cells precede axonal degeneration [22]. These changes that are described in Schwann cells include: cytoplasm edema, glycogen particles aggregates as well as hyperplasia of surrounding basal lamina [22]. 

In the study of De Gregorio et al., it was noticed how complex anatomopathological changes can be in subjects with DN [23]. There is significant reduction in intraepidermal nerve fiber (IENF) density in diabetic mice particularly from 18 weeks of age compared to normal littermates, while in a later period from 26 weeks of age in diabetic mice, a significant increase in TUNEL+ cells in sciatic nerve was observed [23]. The increase of such cells suggests to the greater susceptibility of Schwann cells to apoptosis. Furthermore, at the same age (26 weeks) in diabetic mice, an increase of T lymphocytes in sciatic nerves was also noticed, indicating the increase of the inflammation process [23]. Finally, besides the fact that there was no difference in nerve fiber diameters of sciatic nerve between diabetic and non-diabetic mice, there was an increase in the fibers density particularly in diabetic mice age of 32 weeks [23]. It was shown that there was a decrease in large fibers and increase in small fibers in diabetic mice [23].

In the study of Younger, on sural nerve in human subjects with DN, it was noticed that there was perivasculitis in larger proportion than microvasculitis and necrotizing arteritis (NA) [24]. The presence of microvasculitis and NA might suggest the theory of ischemic axonopathy. However, it seems that microvasculitis and severe perivasculitis have more influence in epineurial ischemia than NA does [24]. Additionally, the study pointed to more frequent primary axonopathy than primary myelinopathy, and the presence of deposits of C3 and C5b-9 complements in the walls of endoneurial microvessels from around two-thirds of tested nerves [24].

## 3. Peripheral Nervous System Dysfunction in Diabetic Neuropathy

The peripheral nervous system has its own specific characteristics from other tissues that are characterized in structure, function and metabolic needs [25]. It should be pointed out that axons and Schwann cells are interdependent [26]. The significant components in saltatory impulse conduction along the myelinated nerve are nodes. Thus, the processes that affects structural and functional parts of these elements including Schwannopathy, axonopathy and nodal degeneration that could be associated with the onset and the progression of PDN. Zenker et al. stressed that nodal degeneration, disjunctions on the axonal-glial levels and altered ion-channel localization might be associated with PDN [25]. Robertson and Sima, in their study on mutant mouse models, described the presence of temporal discrepancy between the occurrence of functional and structural deficits in nerves, suggesting that the origin of the DN might be primarily metabolic [27]. However, it should be noticed that so far, no animal model was produced to be completely comparable with the human DN onset and the progression. 

In DM patients with present neuropathy, metabolic disturbances alter the synthesis of neurotrophic factors both in neurons and Schwann cells that affect the processes of regeneration [28]. Reduced production of nerve growth factor (NGF) and neurotrophin-3 (NT-3) were observed in cultured Schwann cells of diabetic mice [22]. Modifications of Schwann cells functions that might be altered by diabetes, including the changes in the production of signaling molecules as well as impaired receptor-mediated interactions between these cells and basal lamina and the endoneurium, which could affect PNS structure and function [28].

**Figure 1:**
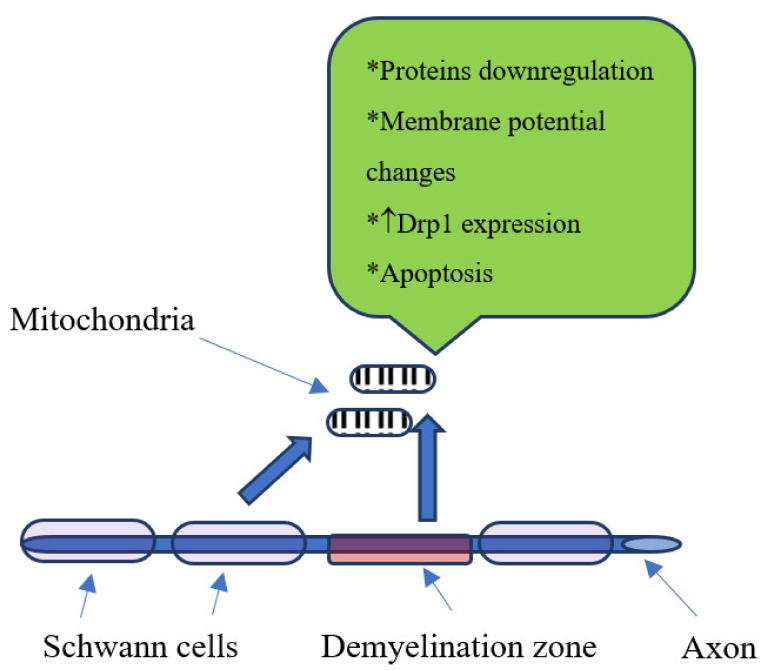
Mitochondrial changes in diabetic neuropathy, ↑-increase.

Another important aspect is the mitochondrial role in neuron and Schwann cells dysfunctions in DM subjects. In diabetic rats there is downregulation of mitochondrial proteins [29]. Furthermore, changes in mitochondrial membrane potential in sensory neurons in diabetic rats were also described [30] as well as downregulation of mitochondrial respiratory chain complex proteins in sensory neurons [31]. Additionally, fragmented mitochondria and an increase in the expression of Drp1 were noticed in sensory neurons of DM subjects [32]. Finally, exposure of mitochondria to the increased concentration of glucose could lead to the apoptosis by releasing into cytosol some apoptosis-induced factors, thus reducing the mitochondrial number in cells [33] (Figure 1).

In previous studies on animal models with DM type 1 it was stressed that induced hyperglycemia is associated with changes in calcium (Ca^++^) homeostasis/signaling pathway and mitochondrial function [34]. The impaired expression and function of Ca^++^ channels are associated with PDN, and Ca^++^ is found to be associated as well with dysfunctions of mitochondria in subjects with sensory DN [34].

## 4. Biochemical Changes in Diabetic Neuropathy

Diabetic neuropathy in subjects with DM could be associated with several conditions: excessive polyol pathway flux, reactive oxygen species formation (ROS), advanced glycosylation end products (AGE) production and inadequate neurotrophic support [26,35]. Furthermore, hyperglycemia was associated also with activation of mitogen-activated protein kinases (MAPK) and upregulation of hexosamine pathway [35,36].

The flux of polyol pathway increases in hyperglycemia in order to metabolize elevated glucose levels and such an increase of the flux is associated with diabetic neuropathy [37]. In diabetes, the polyol pathway is catalyzed by the two enzymes: aldose reductase (AR) and sorbitol dehydrogenase (SD). The localization of AR is noticed in Schwann cells of nerve fibers that are myelinated and in the satellite cells of the dorsal root ganglia [26]. Increased activity of these two enzymes is associated with elevated levels of sorbitol, fructose, NADP^+^ and NADH along with the lower levels of NADPH and NAD^+^ [37]. The conversion of glucose to sorbitol is done by the AR, while the SD‘s role is in the oxidation of the sorbitol to fructose [36]. Additional AR function in DM is the up-regulation of MAPK activity in nerve and dorsal root ganglia [36]. Polyol pathway activation as well as altered protein kinase C activity trigger oxidative stress, thus these mechanisms are associated with the altered cell‘s redox state, leading to the cell injury [35]. Furthermore, previously it was pointed that the lipid peroxidation in nerve cells, as a consequence of polyol pathway flux, can be prevented by inhibiting the AR [26]. 

In diabetic patients, the glucose via nonenzymatic processes forms Amadori products, which are further converted to a more stable substrate called AGE [38]. Further, the AGE attaches to the receptor (RAGE) on the cell surface, initiating a cascade of signal transduction events that lead to the ROS production [38]. 

The role of oxidative stress in pathogenesis of DN was broadly evaluated previously. Increased ROS is considered as one of the factors associated with programmed cell death [39]. This is of particular importance sine ROS were observed in dorsal root ganglia and Schwann cells; thus they might play important role in the DN pathogenesis [39]. Additionally, the oxidative stress affects the mitochondria via several mechanisms that include inhibition of ATP synthesis as well as essential proteins in to mitochondria, and affects mitochondrial membrane permeability by damaging the inner membrane proteins [38]. In a hyperglycemic state, the activation of Nuclear factor-κB (NF-κB), cyclooxigenase-2 (COX-2) mRNA induction as well as COX-2 protein expression are described [40]. The changes associated with the COX-2 upregulation lead to the complex processes resulting in the vasoconstriction and the onset of ischemic conditions in affected tissues as well as ROS production [40].

## 5. Inflammation, Cytokines and Neurotrophins in Diabetic Neuropathy

It is noticed that chronic low-grade inflammation plays an important role in the development and progression of DN [41]. In individuals with DN, increased inflammatory cytokines as well as different levels of certain growth factors were observed [42]. However, it should be mentioned that the inflammation has a more complex role in DN pathogenesis, since it is not only involved in processes that results in nerve damage, but also in process associated with regeneration [43,44].

**Figure 2:**
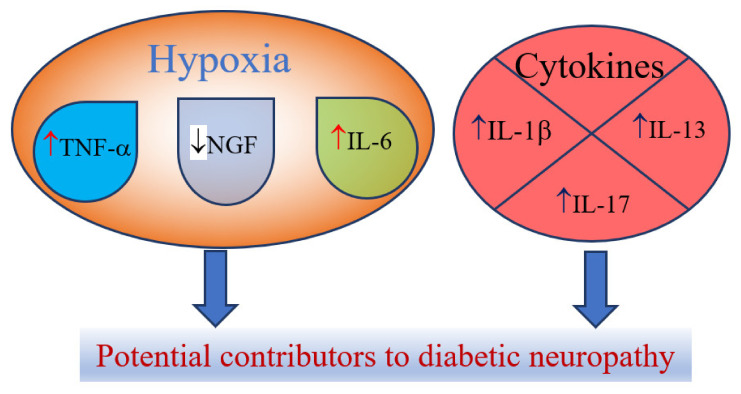
Cytokines and neurotrophins as potential contributors to diabetic neuropathy, ↑-increase; ↓-decrease

Hypoxia or ischemia that are associated with the endothelial injury and or microvascular dysfunctions that lead to the increase in the levels of the certain cytokines including tumor necrosis factor (TNF)-α and interleukin (IL)-6, while such states lower nerve growth factor (NGF) [35]. However, NGF is more closely referred as neurotrophin and plays important roles in the axonal generation stimulation after a neuron injury [45]. Other cytokines could play a role in the pathogenesis of DN, including elevated levels of IL-1β, IL-13 and IL-17 [36] (Figure 2). Previous reports stated that the reduction of IL-1β, IL-6 and TNF-α by genetic deletion or pharmacological inhibition has favorable effects on changes in nerve conduction velocities [43]. As it was stressed in the study of Bönhof et al., IL-6 might have pro-and anti-inflammatory characteristics that could depend on immunological context [43], thus its exact role in the pathogenesis of DN should be studied further. 

## 6. The Role of Dyslipidemia in Diabetic Neuropathy

Previous studies investigated the complex interactions between dyslipidemia and inflammation in diabetic neuropathy [46,47]. In the study of Vincent et al. [47], it was noticed that sorbitol, oxidized lipids as well as poly ADP-ribose polymerase are increased in animal models that were on a high-fat diet, along with the lipoxygenases activation before diabetes development in peripheral nerves. Furthermore, it was stressed that fatty acids with long chains could penetrate the blood-nerve barrier, thus inducing neurogenic inflammation that might lead to the increased expression of TNF-α and IL-6 [46]. Additionally, free fatty acids are shown to have lipotoxicity potential that is mediated via lysosomal dysfunction in cultured neuronal and Schwann cell lines [47]. Moreover, fatty acids produce NADPH and FADPH_2_ through β-oxidation in Schwann cells, dorsal root ganglion and axons [48]. Patients with diabetes—due to the increased accumulation of the acetyl-CoA molecules that are formed in Schwann cells from long-chain fatty acids—convert such molecules into acylcarnitines that affect Schwann cells and dorsal root ganglions, thus such a process contributes to diabetic neuropathy [48]. In a systematic review and meta-analysis of Cai et al., it was stated that the free fatty acids could bind to pyruvate in mitochondria and thus increasing the ROS production [49].

In this environment with increased oxidative stress, the oxidation of cholesterol to oxysterols occurs. The production of oxysterol might be via autoxidation or by the enzymatic oxidation of cholesterol, where those originated by autoxidation are speculated to be the most important oxidative stress biomarkers [50]. In oxysterol metabolism, three main groups of enzymes are described: *oxidoreductases* that are subcellularly localized in the endoplasmic reticulum, mitochondria and cytosol; *hydrolases* that are subcellularly localized in the endoplasmic reticulum; and *transferases* that are subcellularly localized in cytosol [51]. In the study of Ferderbar et al. [52], it was stated that cholesterol oxides could induce apoptosis of several cell types: vascular, endothelial, smooth muscle of the artery as well as T cells and monocyte macrophages. Moreover, they can act proinflammatory by modulating the synthesis of cytokines, adhesion molecules and growth factors [52]. Additionally, oxidized low-density lipoproteins (LDL) are binding to oxidized LDL receptor 1 (LOX1), Toll-like receptor 4 (TLR4) and RAGE contributing to the inflammation processes and increase of ROS [48].

## 7. Genetic Basis of Polyol Pathway

The complex role of the AR gene in the etiopathogenesis of complications in individuals with DM could be referred to the fact that AR gene expression might be induced by methylglyoxal (MGO), advance glycation end-product (AGE) and oxidative stress in a hyperglycemic state [53]. Previous investigations of DN pathology on rodent models and humans pointed that mice do not tend to have higher loss of nerve fibers even long-term in DM [53]. Thus, it might be assumed that other factors could be involved in the progression of DN, while it is suggested that AR could be involved in the early stage of DN [53].

The AR is encoded by the *ALR2* gene, while SD, the second enzyme in polyol pathway, is encoded by the *SDH* gene [54]. In the study of Sivenius et al. for a group of patients with DM type 2, it was noticed that the-106C/T polymorphism of promoter region of *ALR2* gene is associated with a decrease in the nerve conduction velocities of the motor peroneal nerve and a lower amplitudes of the tested sensory nerves versus subjects with-106C/C genotype [55]. Heesom et al., in the study of patients with insulin dependent DM, found the association between the DN and another polymorphism located at the 5‘, upstream regulatory region of *ALR2* [56]. This is the 5′-(CA)n microsatellite polymorphism represented with more than 10 alleles; two major alleles are Z-2 and Z+2, whereby Z corresponds to 24 CA repeats. The group of patients with DN had significantly decreased frequency of the Z+2 allele [56], while it was noticed that the Z-2 allele is associated with the increased susceptibility of diabetic complications in both types of DM [53]. It should be pointed that these markers are located outside of the protein coded region of AR gene, indicating that they should not directly affect the enzyme structure and function [53]. Thus, the possible mechanisms of action for some of these alleles could be enhancement of AR gene expression or the transcription of the one [53]. 

Considering the SD role in DN, it should be noticed that the nerve sorbitol levels in SD deficient mice are not suggestive for DN susceptibility [53].

## 8. Other Genetic Risk Factors for Diabetic Neuropathy

In their review, Witzel et al. contributed to the creation of a genetic-metabolic model that should help to elucidate the cause of diabetic neuropathies, evaluate a patient’s risk profile and facilitate preventative and targeted treatment for the individual [1]. After evaluation of published candidate genetic markers in different forms of DN, the authors summarized common genetic risk factors. Polymorphisms in genes *ACE* (angiotensin-converting enzyme), *APOE* (apolipoprotein E), *MTHFR* (methylene tetrahydropholate reductase), *NOS3* or *ENOS* (nitric oxide synthase 3 or endothelial nitric oxide synthase) and *VEGF* (vascular endothelial growth factor) have been shown to contribute to DPN as well as DN. In all of these genes, the minor polymorphic allele is associated with DN [1]. It was speculated that having in mind functions of these genes, it is possible that the same variants also contribute to UN (uremic neuropathy) and CAN (cardiac autonomic neuropathy). Considering candidate genes that have been implied in but not significantly associated with the diabetic neuropathies, this number of potentially genetic risk factors could be larger. Authors emphasized that all of the listed genes are involved in key molecular pathways that have been linked to diabetes and its complications, mentioned above [57,58,59]. 

Very recently, Zhao Y et al. performed a systematic review, meta-analysis and trial sequential analysis (TSA) of the association between genetic polymorphisms and DN risk [2]. They conducted a systematic review of more than 1350 articles and publications reporting on 136 polymorphisms in 76 genes. More than 100 meta-analyses on 36 studies were performed involving 12,221 subjects to derive pooled effect estimates for analyzed polymorphisms. 

Data for the most important genes processed in the mentioned studies will be presented in the following text.

The ACE is an enzyme and vasoconstrictor playing a crucial role in the renin–angiotensin system by doing conversion of angiotensin I to angiotensin II. As a reminder, it is well documented that angiotensin II is involved in regulation of glucose and insulin levels, and among other effects that induce oxidative stress, inflammation and vascular changes [4,44]. The *ACE* gene insertion/deletion (I/D) polymorphism is widely studied because this variant has a regulatory role on gene expression and itself is responsible for about 46% of enzyme serum variance [1]. The *ACE* I allele is defined by presence of 287bp Alu sequence in intron 16, while in the D allele this sequence is deleted; D allele and DD genotype are associated with elevated ACE enzyme levels [29]. A number of original investigations and several meta-analyses confirmed the role of *ACE* I/D polymorphism in DN, indicating D allele as a risk factor, particularly for DPN [4,60,61,62,63]. In addition, association of D allele with renal disease, as well as with blood pressure variations in DM, indicates its role in UN and CAN, respectively. On the contrary, the *ACE* II genotype is assigned as protective regarding DN. The *ACE* I/D polymorphism has been considered as potential pharmacogenetic marker because it could affect efficiency of ACE inhibitors, angiotensin receptor blockers and statins, which are widely used in DM. However, the clinical benefit of routine *ACE* I/D genotyping in this purpose is still controversial [1,2,4]. 

APOE is a cholesterol transporter and LDL receptor ligand involved in lipid metabolism, nerve repair and regeneration. Well-known allelic variants e2, e3 and e4 in *APOE* gene are defined by two functional single nucleotide polymorphisms (SNPs), rs429358 C/T and rs7412 T/C, which cause amino acid changes 112Cys/Arg and 158Arg/Cys, respectively, at the protein level [1,2,44]. *APOE* gene variants have an impact on protein function trough the isoforms E2, E3 and E4, which differ in charge and stability. In general, the e2 allele is associated with higher circulatory APOE and lower levels of LDL cholesterol, while e4 allele shows opposite effects. The *APOE* genotype is associated with disorders of both the central and peripheral nervous system, and e4 allele is assigned as a risk variant for neuropathology. Regarding the role of *APOE* in DM and DN, studies show some population specificities. Meta-analyses confirmed e4 allele as a risk variant for DPN, and there are data about particularly severe DPN in e4 carriers also [1,2,64,65]. In addition, both clinical studies and animal models suggest *APOE* as candidate gene for CAN risk. Besides, pharmacogenetic evaluation of *APOE* is a very current topic. It has been found that the antidiabetic drug Metformin enhances *APOE* expression, which consecutively stimulates nerve regeneration. Some studies showed association of *APOE* e4 form with a worse response to statins, but a better response to life style changes in DM patients. These results are still inconsistent but could have effect on various aspects of DM and DN monitoring and control [1,2]. 

MTHFR is an enzyme in folate cycle and homocysteine metabolism with a role in neurotransmitter production, protein synthesis, immune response and inflammation. MTHFR plays important role in remethylation of homocysteine to methionine and its lower activity is linked to hyperhomocysteinemia, which has damaging effects on blood vessels, lipid metabolism, neuronal function, etc. [1,4,6]. In DM, high blood homocysteine is associated with neurovascular complications. Frequently studied polymorphisms *MTHFR* 677C/T (rs1801133) and 1298A/C (rs1801131) lead to amino acid changes 222Ala/Val and 429Glu/Ala, respectively, and affect enzyme structure and function. After conflicting results of numerous studies, meta-analyses showed significant association of DN, including DPN and CAN, and *MTHFR* 1298A/C variant, but no clear association with 677C/T [1,2,4,66,67,68,69]. It is important to emphasize that about 35% of variations in homocysteine levels are due to folate and vitamin B12 levels, and that diet may diminish negative genetic effects, at least to some extent. This is of particular interest in assessment of *MTHFR* variants as pharmacogenetic markers. It has been found that *MTHFR* polymorphism affect response to various drugs, including antidiabetic drug Metformin. As Metformin usage leads to vitamin B12 deficiency, DM patients at treatment who have risky *MTHFR* genotypes should take vitamin supplements [1]. 

NOS3 (eNOS) regulates NO circulating levels by production of NO from L-arginine, maintains endothelial cell function and homeostasis. In the *NOS3* gene, three important polymorphisms are: promoter variant-786T/C (rs2070744), functional SNP 894G/T (rs1799983) and variation in tandem repeats in intron 4, known as 4a/b. Several studies detected association of DN and DPN occurrence and progression with all three *NOS3* polymorphisms, while for UN are CAN results are negative or not consistent [1,70,71]. NOS3 is interesting therapeutic target, because of its association to neuropathic pain and inflammation, and mentioned variants are potential pharmacogenetic markers [1]. 

VEGF is chemokine that modulates vascular permeability, regulates angiogenesis and contributes positively to neurogenesis [1,2,44]. Related to *VEGF* gene there are two polymorphic sites, rs6921438 and rs10738760, which contribute to about 50% of VEGF level variation. Association of *VEGF* related polymorphisms and DPN are observed, but a significant link to CAN and UN is not clearly established. Further investigation about contribution and pharmacogenetic effects of these genetic variants in DNs is needed [1,2,72].

Considering anti-oxidative mechanisms in DN, variants in genes encoding ubiquitous enzymes that catalyze the removal of hydrogen peroxide, or that quench reactive molecules have been widely studied. Meta-analyses confirmed association of polymorphism 599C/T (rs1050450) in the gene *GPx-1* (glutathione peroxidase 1) and-262C/T in the gene *CAT* (catalase) with DN, while no significant association was observed with null/present variants in *GSTM1* and *GSTMT1* (glutathione S-transferases M1 or T1) [2,73].

Contemporary genomic studies are also conducted in the field of DN. Meng et al. published results of a genome-wide association study, which was undertaken in Scotland. They found that chromosomal loci 1p35.1 and 8p21.3 were associated with neuropathic pain in DPN, with a more prevalent association in women [74]. However, a noticed weakness of this study is about criteria for painful-DPN. Future well-designed studies are needed to confirm these results. 

In Table 2, the most relevant gene polymorphisms associated to DN were presented.

## 9. Epigenomic Modifications in Diabetic Neuropathy

**Figure 3:**
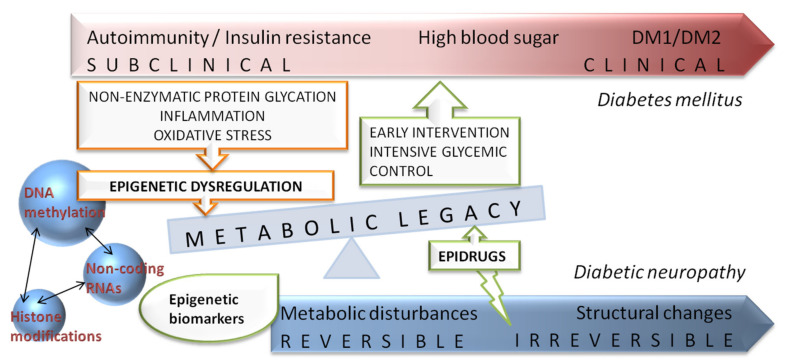
Schematic representation of diabetes and diabetic neuropathy progression with integrated view of potential benefits of epigenetic related biomarkers and novel epidrugs on “metabolic memory” or “metabolic legacy” phenomenon.

Major epidemiological studies revealed that an early intensive glycemic control is able to decrease the long-term risk of diabetic complications, the phenomenon termed as “metabolic memory”. Although the mechanism of “metabolic memory” is not completely understood, recent studies implicated that continuing metabolic changes are established trough oxidative stress, non-enzymatic glycation of proteins, chronic inflammation as well as dysregulation of epigenetic mechanisms [75]. Epigenetic modifications are defined as heritable changes in gene expression patterns without alterations in the DNA sequence, emerging from genome–environment interactions [76]. Mounting evidence suggests a key role of different epigenetic mechanisms such as DNA methylation, non-coding RNAs and post-translational histone modifications in “metabolic memory” and the risk of developing diabetic complication [77] (Figure 3).

**DNA methylation** is a stable and reversible attachment of methyl (CH3) group to a cytosine in palindromic sites in DNA, named CpG islands. Those sites are usually located near or in promoter and regulatory regions allowing the family of enzymes, DNA methyltransferases (DNMTs), to regulate gene function trough methylation process [78]. DNA methylation is regulating gene expression and has an important role in many biological functions including disease susceptibility [79]. Genome-wide methylation analysis of samples collected 16–17 years apart from the same diabetic patients revealed a persistency of DNA methylation over time at key genomic loci associated with diabetic complications [80]. Hyperglycemia is changing DNA methylation status inducing alterations in gene expression related to PDN and significantly reduced methylation of whole genomic DNA in white blood cells represents a potential biomarker for PDN [81]. Additionally, new evidence has pointed out that different DNA methylation patterns in genes related to nerve regeneration and functionality have an important role in the development of cardiovascular autonomic neuropathy (CAN) in patients with DM type 1 [82]. For instance, an increased methylation, correlated with the decreased expression, was observed in the ninjurin 2 (*NINJ2*) gene. NINJ2 protein in Schwann cells is promoting nerve regeneration after injury [83], therefore downregulating *NINJ2* expression may contribute to neuropathies development. Decreased methylation of genes involved in neuronal polarization (BR serine/threonine kinase 2 (*BRSK2*)) was also observed, but not confirmed. An interesting finding from the same study was a strong hypomethylation of the 5′UTR region of the claudin 4 (*CLDN4*) gene. Although it is supposed to promote the attachment of the transcriptional factor, it was associated with the significant downregulation of gene expression. The authors suggested that the involvement of other epigenetic mechanisms may be involved in decreasing gene expression [82,84]. Promising findings from genome-wide profiling of DNA methylation in sural nerves of patients with differences in progression of PDN is linking specific DNA methylation profiles with PDN progression through pathways related to nervous system development and/or axon guidance (netrin-4 (*NTN4*) and dihydropyrimidinase like 2 (*DPYSL2*) genes), glycerophospholipid metabolism (phospholipase A2 and phosphatidylserine decarboxylase) and MAPK signaling [85]. Moreover, integrated analysis of the sural nerve methylome and transcriptome identified DN candidate genes and pathways (immune response, ECM regulation and PI3K-Akt signaling) [86]. Guo et al. also observed multiple differentially methylated CpGs within regulatory regions that may affect the expression of lncRNAs or miRNAs. Highest methylation difference is observed in miR3138 whose target is erb-b2 receptor tyrosine kinase 4 (*ERBB4*), a gene previously associated with progression of PDN [87].

**MicroRNAs (MiRNAs)** are group of evolutionarily conserved, regulatory non-coding RNAs comprise of less than 200 nucleotides in length (about 20) that are complementary to 3′-UTR of different messenger RNAs (mRNAs). Binding miRNA with targeted mRNA disrupts translation and leads to mRNA cleavage [88]. The role of miRNAs different expression patterns in multifactorial diseases like DN is a rapidly evolving field because of their potential use as biomarkers of disease progression and response to therapy, as well as promising targets for developing epidrugs [89,90]. Study in patients with diabetic neuropathy showed an increased expression of miR199a3p and subsequently downregulation of extracellular protein Serpin E2 in disease progression [91]. Increased expression of miR9 in PDN rat model upregulated CALHM1, a protein involved in DN pathophysiology [92]. Conversely, several studies in diabetic animal models identified decreased miR25, miR146 and miR190a5p expression [93,94,95,96] (Table 3). Besides studies focused on alternations in miRNAs expression, it has been shown that polymorphisms in miRNAs genes are affecting the maturation process or target recognition and binding, and may be involved in pathological processes in different diseases [97]. Genetic variants in *MIR128A*, *MIR146A*, *MIR27A* and *MIR499A* genes have been associated with the risk of PDN and CAN in Italian patients with DM type 2 [98,99]. Furthermore, the association between decreased number of mtDNA copies and polymorphism in *MIR499A* gene has been recently described in DM type 2 patients, particularly with DN, suggesting a possible role of oxidative stress in the development of DN [100].

**Long non-coding RNA (LncRNAs)**, RNAs with more than 200 nucleotides in length, have a regulatory function in numerous metabolic processes and pathways in the nucleus and cytoplasm [101] and it is not surprising that several recent studies revealed specific lncRNAs (48+, LncRNANONRATT021972 and BC168687) as players in pathogenesis of DN and neuropathic pain [102,103,104]. Their role is further confirmed with findings that small interference RNAs (siRNA) for specific lncRNAs in diabetic rat models are attenuating PDN potentially through reduced production of inflammatory factors [105,106,107,108,109]. Genome-wide expression differences of lncRNAs between DM patients with and without PDN were identified using microarray analysis. The lncRNA/mRNA coexpression network in PDN patients indicated involvement of the neurotrophin-MAPK signaling pathway. Several lncRNAs (CCNT2-AS1, RP1-249H1.2, CTD-3239E11.2, RP11-51B23.3, STAM-AS1 and LINC00629) were indicated as potential interplayers in the abovementioned signaling pathways and additional, previously reported diabetes-associated lncRNAs (MALAT1, H19, PVT1, MEG3, MIAT and MIR143HG) followed trends observed in the microarray analysis [110] (Table 3). Results from similar animal studies also revealed enriched pathways including retrograde endocannabinoid signaling, amphetamine addiction and dopaminergic synapse [111], as well as PI3K-Akt, MAPK, PPAR and Toll-like receptor signaling pathway [112].

**Table 3:** Summary of recent studies of non-coding RNAs expression in diabetic neuropathy pathogenesis.

Non-Coding RNAs	Potential Mechanism in PDN	Model System	References
**MiRNA**			
miR199a3p	Increased expression, Serpin E2 downregulation	DM Patients (peripheral blood)	[91]
mir9	Increased expression, CALHM1 upregulated	PDN rat model (spinal dorsal horn)	[92]
miR25	Decreased expression, regulation of oxidative/nitrative stress	Diabetic mouse (sciatic nerves)	[93]
miR146	Decreased expression, negatively correlated with the levels of IL-1β, TNF-α and NF-κB	T2DM and PDN rat	[94,95]
miR190a5p	Decreased expression, SLC17A6 upregulated	Diabetic mouse	[96]
**LncRNA**			
48+	Increased expression, P2X3 Receptor upregulated	Diabetic rat (dorsal root ganglia)	[102]
	Increased expression, P2X7 Receptor upregulated	Diabetic rat (superior cervical ganglia)	[107]
NONRATT021972	Increased expression, neuropathic pain higher scores/TNF-a upregulated	DM2 Patients (peripheral blood)/diabetic rat	[103]
	Increased expression, P2X7 Receptor upregulated	PC12 Cells	[106]
		Diabetic rat (dorsal root ganglia)	[108]
BC168687	Increased expression, TRPV1 Receptor upregulated Increased expression, P2X7 Receptor upregulated	Diabetic rat (dorsal root ganglia)	[104,109]
CCNT2-AS1	Neurotrophin-MAPK signaling pathway affecting	DM/PDN patients (peripheral blood)	[110]
RP1-249H1.2			
CTD-3239E11.2			
RP11-51B23.3			
STAM-AS1			
LINC00629			
MALAT1 (NEAT2)	Increased expression in DM and PDN patients, affecting multiple physiological processes		
MIR143HG	Decreased expression in PDN patients		
PVT1			
H19	Decreased expression in PDN patients VDAC1 downregulation in diabetic cardiomyopathy	DM/PDN patients (peripheral blood) Cultured neonatal rat cardiomyocytes	[110,113]

DM–diabetes mellites PDN–peripheral diabetic neuropathy; T2DM–type 2 diabetes mellitus.

**Post-translational histone modifications** (acetylation, methylation etc.) are dynamic processes regulating DNA wrapping in nucleosome formation and chromatin organization. The most prominent mechanism, histone acetylation, is under the regulation of two groups of enzymes: histone acetyltransferases (HATs) and histone deacetylases (HDACs) [114]. Inhibitors of HDACs interfere with the epigenetic process of acetylation and have a role in neuropathic pain [115], peripheral nerve injury–induced neuropathic hypersensitivity [116], but also in diabetic retinopathy and nephropathy [117,118]. HDACs represent potential therapeutic targets in the treatment of diabetic peripheral neuropathy. Studies of the peripheral nerves of diabetic animal models and in vitro high glucose–cultured Schwann cells (RSC96) revealed that STAT3 and HDAC1 are promising targets in PDN prevention via the improvement of autophagy [119]. 

## 10. Potential Treatment Targets

It is evident that DN is a complex condition, thus potential treatment management should target numerous pathogenic mechanisms and abnormalities that are shown to be related with DN onset and progression. In the statement of the American Diabetes Association published by Boulton et al. [120], certain aldolase reductase inhibitors were shown to be effective in randomized clinical trials (RCT) in reduction of nerve sorbitol, thus affecting an increased polyol pathway. α-Lipoic acid for reduction of oxygen free radicals, as well as angiotensin converting enzyme (ACE) inhibitors and prostaglandin analogs for increase of nerve blood flow were shown to be effective in RCTs as well [120]. Previously in this paper, the potential roles of ACE inhibitors and metformin were described.

Moreover, it should be noted that in treating DM as a condition, the complications including DN might be reduced or even prevented. In the meta-analysis of Vujkovic et al. [121], numerous drug-gene relationships in DM type 2 patients were described. Furthermore, there are several drugs (sulphonylureas, metformin and thiazolidinediones) that are widely used in the treatment of patients with DM type 2. In the study of Pollastro et al., it was noted that there is an association of certain polymorphisms in several genes (ABCC8, KCNJ11, TCF7L2, CYP2C9, IRS1 and CAPN10) and sulphonylureas responsiveness [122]. The presence of polymorphisms (R61C, G401S, 420del and G465R) in the SLC22A1 gene, as well as certain SLC47A1 and ATM gene variants are associated with metformin responsiveness [122,123]. 

Drugs currently available for the treatment of neuropathic pain show only modest improvement in symptoms, but sometimes significant side effects. In order to improve therapeutic options for DN, recent studies have analyzed genes encoding for voltage-gated sodium channels and their role in neuropathic pain. One good example is the SCN9A gene coding Nav 1.7 sodium channel, which is involved in pain signaling. Investigations have identified SCN9A gain of function mutations in idiopathic small fiber neuropathy [124] and in painful DPN [125]. In the study of Blesneac et al., rare Nav 1.7 variants were found in 10 out of 111 patients with painful-DPN, but in none of 78 respondents with painless-DPN [126]. In addition, subjects with Nav 1.7 variants showed shorter duration of diabetes and more severe pain. These results are interesting from a clinical point of view because carriers of these genetic variants treated with the anticonvulsant lacosamide had significantly improved pain compared with a placebo [127]. In the era of precision medicine, more similar relevant data are expected. 

Additionally, development of novel therapeutics for DN is one of the main focuses in recent human and animal studies. The previously mentioned chemokine, VEGF, is considered as prospective new therapeutic treatment for DN, even for a gene therapy approach. Some studies indicated that upregulation of VEGF could have neuroprotective and neurotropic function, and so could be used for the treatment of DN. However, others showed pathological angiogenesis and vascular permeability after VEFG administration, with consecutive worsening of DN and other DM complications [57]. Such contradictory effects could be explained by different roles of VEGF in the DM onset and progression, as well as by differences in DM and non-DM cases.

As a potential novel epidrug, trichostatin A (TSA) treatment is targeting histone deacetylases and reversing reduced expression of brain derived neurotrophic factor (*BDNF*) in diabetic mice [128]. Another innovative approach in epidrug development is the application of pharmaceutical nanocarriers with different miRNAs. MiR146a5p, was used to create nanoparticle–miRNA-146a-5p polyplexes (nano-miR-146a-5p) with protective effect of on peripheral nerves in the DPN rat model, probably through the inhibition of expression of proinflammatory cytokines [129]. 

## 11. Conclusions

Although PDN has a high prevalence and strong impact on the quality of life of DM patients, it is still underdiagnosed, and current therapies are not fully efficient in the majority of cases. Accumulating evidence of epigenetic mechanisms underlying PDN pathogenesis may lead to identification of novel drug targets and development of epidrugs in the era of precision medicine. 

## Figures and Tables

**Table 1:** Characteristics of different types of neuropathies in diabetic patients.

	Type of Neuropathies due to Fiber Size		Autonomic Neuropathy [12,13,14,15,16,17,18,19,20]		
	Small Fiber [11,12]	Large Fiber [12]	Systems	Presentation	Possible Pathogenesis and Origin
Motor deficits	P	+	Cardiovascular	Myocardial infarctions	Coronary artery disease
				Nocturnal hypertension	Sustained adrenergic activity during sleep
				Orthostatic hypotension	Efferent sympathetic vasomotor denervation
				Tachycardia	Vagal cardiac neuropathy
Sensory loss	+	+		Sudden death	Modified perception of myocardial ischemia; impaired hemodynamic response to cardiovascular stresses; cardiac arrhythmias; impaired sympathetic–parasympathetic cardiac innervation balance
Pain	+; Sharp;of C fiber type	+	Gastrointestinal	Gastroparesis	Impaired vagal activity and intrinsic enteric autonomic nerves, reduced nerve fiber amount in antral biopsies, loss of neuronal nitric oxide synthase (nNOS)
				Diarrhea	Visceral hypersensitivity
				Constipation	Not fully understood; altered secretion of gastrointestinal hormones
				Fecal incontinence	Incompetence of anal sphincter or ↓ rectal sensation
Vibration sensation	↓	↓	Genitourinary	Bladder dysfunction	Afferent and efferent autonomic nerves dysfunction; bladder smooth muscle dysfunction; and urothelial abnormalities
				Erectile dysfunction	Morphological alterations of autonomic nerve fibers
Thermal sensation	↓	↓		Ejaculatory failure	Sympathetic nervous system dysfunction
Tendon reflex	P(∗)	Depressed		↓ Sexual desire in females	Sexual dysfunction in diabetic females might be related more to psychogenic factors, probably due to the disease burden
Origin	Dorsal root ganglion			↑ Pain during intercourse in females	
Presentation	Length-dependent **	Various sensory and motor signs	Sudomotor	Loss of sweating, dry cold skin	Impaired postsympathetic cholinergic nerve fibers activity, which release acetylcholine
Autonomous symptoms	+/−; impaired blood flow	↑ blood flow			

P–preserved; +-present; ↓-decreased; *-might be decreased in elderly with small fiber neuropathy; **-mostly; +/−-might be present in around half of the patients.

**Table 2:** The most relevant gene polymorphisms associated to diabetic neuropathy.

Gene/Locus	Polymorphism	Risk Allele	Mechanism	References
*ALR2*	-106C/T	T	Polyol pathway, oxidative stress	[55]
	5′-(CA)n	Z-2 (CA_22_)		[53,56]
*ACE*	I/D (intron 16)	D	oxidative stress, vascular changes	[4,60,61,63]
*APOE*	e2, e3, e4	e4	lipid metabolism, nerve repair and regeneration	[1,2,64,65]
*MTHFR*	677C/T	T	hyperhomocysteinemia, lipid metabolism	[1,2,4,66,67,68,69]
	1298A/C	C		
*NOS3*	-786T/C	C	vascular changes, oxidative stress	[1,70,71]
	894G/T	T		
	4a/b	4a		
6p21.1 (*VEGF–related)*	rs6921438 A/G	A	vascular changes	[1,2,72]
9p24.2 (*VEGF–related)*	rs10738760 A/G	A	vascular changes	
*GPx-1*	599C/T	T	oxidative stress	[2,73]
*CAT*	-262C/T	T	oxidative stress	[2,73]
